# Magnetic resonance arthrography for femoroacetabular impingement surgery: is it reliable?

**DOI:** 10.1007/s10195-013-0227-1

**Published:** 2013-02-09

**Authors:** Alessandro Aprato, Alessandro Massè, Carlo Faletti, Angiola Valente, Francesco Atzori, Maurizio Stratta, Narlaka Jayasekera

**Affiliations:** 1 Pelvic Unit, Orthopaedic Department, II Faculty of Medicine, San Luigi Hospital of Orbassano, University of Turin, Regione Gonzole n.10, 10043 Turin, Orbassano Italy; 2 Department of Radiology, CTO Hospital of Turin, Turin, Italy; 3 The Richard Villar Practice, Spire Cambridge Lea Hospital, Cambridge, UK

**Keywords:** Femoroacetabular impingement, ArthroMRI, Hip

## Abstract

**Background:**

Magnetic resonance arthrography (MRA) is commonly used to demonstrate injury to the labrum and hyaline cartilage in patients with femoroacetabular impingement (FAI). The purpose of this study was to assess the diagnostic correlation between MRA and findings at arthroscopic and open surgery.

**Materials and methods:**

MRA reports of 41 hips with symptomatic FAI were reviewed and compared with subsequent intraoperative findings (*n* = 21 surgical dislocations and *n* = 20 therapeutic hip arthroscopies). Each case was assessed for the presence of a cam deformity, a cartilage lesion of the femoral head, an os acetabuli, an injury to the labrum and injury to the acetabular cartilage. Results were collected prospectively in a cross-table and analysed retrospectively for sensitivity, specificity, positive predictive value (PPV) and negative predictive value (NPV).

**Results:**

The sensitivity, specificity, PPV and NPV in the presence of reported cam-type deformity or an os acetabuli were 100 %. In the presence of cartilage lesions of the femoral head, the values were 46, 81, 55 and 73 %, respectively. For labral tears, the values were 91, 86, 97 and 67 %. In the presence of acetabular cartilage injuries, the values were 69, 88, 78 and 81 %, respectively.

**Conclusions:**

MRA appears to be an efficacious imaging modality in the evaluation of labral tears, cam-type impingement lesions and os acetabuli of the hip. MRA is less efficacious in the diagnosis of cartilage abnormalities in the hip, both femoral and acetabular. Researchers should focus on further improvements in imaging techniques in order to give reliable preoperative information to the surgeon.

## Introduction

Femoroacetabular impingement (FAI) is a recognised cause of hip pain in young adults and a precursor to osteoarthritis [1]. Abnormal femoral and acetabular morphology and lesions of the acetabular labrum and cartilage are common features in FAI. Surgical treatments for FAI include arthroscopy, mini invasive surgery and surgical dislocation [2]. Although there is no consensus on the correct indication for each treatment, surgical treatment should be directed by a precise preoperative diagnosis. Preoperative magnetic resonance arthrography of the hip [3] is useful in guiding surgery for an injured labrum and hyaline cartilage. Introduction of fluid into the hip joint causes the joint to distend and allows for better separation of intra-articular structures, thus allowing for better delamination of pathology at magnetic resonance imaging (MRI). Gadolinium enhancement further improves delamination of pathology involving the hip joint due to its ability to take advantage of the T1-shortening effect and extension of the dynamic range of contrast seen at MR imaging. Furthermore, the use of radial imaging has been described in the literature as a way to better visualise the acetabular labrum [4].

Although most surgeons agree that MRA plays a crucial role in guiding the best treatment for FAI, to our knowledge only a few studies have compared intraoperative findings with the findings of preoperative MRA. The purpose of this study was to assess the diagnostic accuracy of MRA reporting in the detection of these lesions.

## Materials and methods

At our institution from 2008 to March 2010, all patients with a clinical diagnosis of FAI (inclusion criterion: a positive impingement test, defined as pain and decreased range of motion with flexion, adduction, and internal rotation of the hip) were evaluated with plain anteroposterior pelvic and lateral radiographs of the hip preoperatively. Patients with radiographic signs of osteoarthritis were excluded (Tonnis [5] grade >1). Informed consent was obtained from all patients enrolled onto this study. The study was performed in accordance with the ethical standards of the 1964 Declaration of Helsinki, as revised in 2000 and was approved by the Institutional Review Board.

Based on radiographic imaging [6], patients were attributed surgeons for the different types of FAI according to the following criteria:

1. Cam: alpha angle (as described by Tannast [6]) was >50°, and no other sign of FAI was present.
2. Local pincer: an os acetabuli or a prominent anterior wall was present (positive crossover sign with a negative posterior wall sign) and the alpha angle was <50°.
3. Pincer: there were signs of global acetabular deformity (positive crossover and posterior wall signs) or coxa profunda and an alpha angle of <50° were present.
4. Mixed: a local or global deformity was associated with a cam.

All enrolled patients were evaluated with MRA after obtaining their consent. All participants were informed of the risks, limitations, complications and benefits of the procedure. Under fluoroscopic guidance, into the superolateral portion of the coxofemoral joint, at the point of passage between the femoral head and neck, 2–3 ml of Iopamidol (Iopamiro 300^®^, Bracco, Milan, Italy) were introduced through a spinal needle as radiopaque marker to locate the joint capsule.

The paramagnetic contrast agent was then introduced: the acid salt gadopentetic dimeglumine (Magnevist^®^; BayerSchering, Leverkusen, Germany), at a concentration of 0.002 mmol/ml (2 mmol/l), leading to a dose of 0.2 ml/kg of patient body weight. The MR procedure was started immediately after intra-articular administration of the paramagnetic contrast agent. We used 1.5 T equipment at our Department of Diagnostic Imaging (Excite, GE Medical Systems, Milwaukee, WI, USA). We applied traction of 5 kg to the ipsilateral ankle compared to the articulation coxofemoral joint in order to obtain a paraphysiological diastase of the articular heads and to improve the spread of the intra-articular contrast agent. Using a “TORSOPA” or “body” coil, the study protocol provided axial proton-density weighted images with saturation of the signal for adipose tissue (TR 1,300 ms, TE 51 ms, FOV of 22 cm, matrix of 416 × 288, slice thickness of 4 mm, scan time 3′39″), a T1-weighted scan in the coronal plane (TR 140 ms, TE 3.6 ms, FOV of 22 cm, matrix of 416 × 320, slice thickness of 3 mm, scan time 4′31″), sagittal proton density weighted images with saturation of the signal for adipose tissue (TR 1,300 ms, TE 52 ms, FOV of 24 cm, matrix of 256 × 224, slice thickness of 4 mm, scan time 4′10″), and a radial proton density weighted FSPGR with saturation of the signal for the adipose tissue (TR 2,080 ms, TE 51.16 ms, FOV of 20 cm, matrix of 256 × 320, slice thickness of 3 mm, scan time 4′06″). In addition to the standard MRI protocol, radial PDW sequences (TR 2,000 ms, TE 15 ms, FOV of 260 × 260 mm, matrix of 266 × 512, section thickness of 4 mm, 16 slices, scan time 4′43″) were oriented along the axis of the femoral neck. Magnetic resonance arthrography studies from external institutions were not included in the analysis because, in general, they showed poor spatial resolution, as they had been performed with a large field of view or with low-field-strength magnets.

The presence/absence of a cam-type deformity, a cartilage lesion of the femoral head, an os acetabuli, an injured labrum or an acetabular cartilage lesion was evaluated and recorded. A cam-type deformity was considered to be present when the alpha angle [6] was >50° at MRA. Cartilage delamination was considered to be present when hypointense areas in the acetabular cartilage seen on intermediate-weighted fat-saturated or T1-weighted images were present, or when the following two criteria were met: (1) at least two consecutive slices in the same plane or in the same location in two different planes showed focal discontinuity of cartilage and fluid located between the articular cartilage and subchondral bone plate, and (2) the area of abnormal cartilage was not completely detached from the adjacent cartilage [7]. Labral tears were considered positive if staged worse than grade IIA according to the Czerny classification [8]. Os acetabuli was defined as the presence of heterotopic bone on the acetabular rim.

All of the X-ray images and the MRA of these hips were analysed by two subspecialty-trained musculoskeletal radiologists, each with more than 5 years of experience in MRA of the hip joint. The analysis was performed jointly and before surgery.

Based on the clinical and radiological findings, patients were treated with either surgical dislocation (group A) or arthroscopy (group B) according to these criteria: arthroscopy was used in cases of cam-type deformity, focal anterior pincer, or mixed-type FAI (where the cam-type deformity was associated with a focal anterior pincer), while surgical dislocation was performed in cases of pincer or mixed type (where the cam deformity was associated with a coxa profunda or a pincer which, according to our experience, and as alluded to in the literature [2], can be difficult to treat arthroscopically).

The presence/absence of a cam-type deformity was detected intraoperatively in group A using a template, while a dynamic intraoperative assessment for impingement was utilised in group B. Cartilage lesions were assessed intraoperatively and considered positive in both groups if staged greater than grade 2 according to the Outerbridge classification and/or any signs of delamination were detected [9]. Injuries to the labrum were assessed intraoperatively by probing the labrum during arthroscopy and open surgery.

**Fig. 1:**
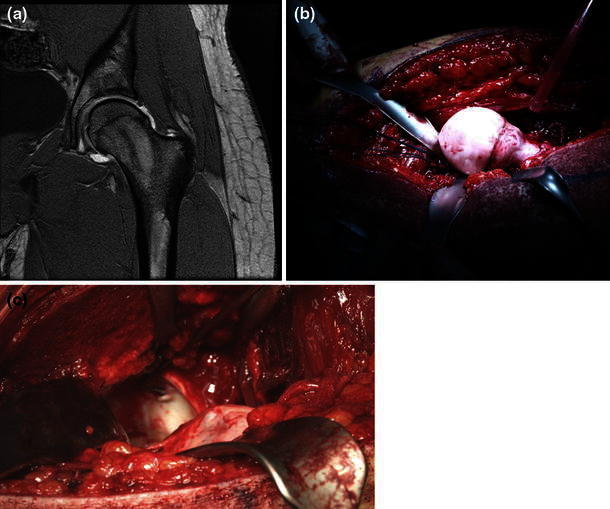
MR arthrogram (**a**) shows a cam without an evident cartilage lesion; intraoperative pictures (**b** and **c**) show a cam with an acetabular cartilage lesion detected through hip dislocation

**Fig. 2:**
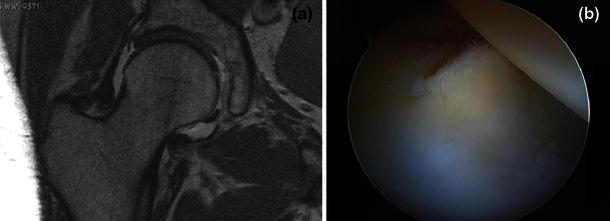
MR arthrogram (**a**) shows a labral tear associated with an acetabular cartilage lesion; intraoperative arthroscopic images (**b**) confirm the report

All (positive and negative) intraoperative findings were recorded (Figs. 1, 2).

Magnetic resonance arthrography reports were compared with intraoperative findings for both groups. For each group and for every item, results were collected in a cross-table and the sensitivity, specificity, positive predictive value (PPV), negative predictive value (NPV) and accuracy were calculated.

### Statistical analysis

All patient data were recorded in a custom-made database and analysed using a commercial software package (TexaSoft, WINKS SDA software, 6th edn., Cedar Hill, TX, USA, 2007). Intraoperative findings at surgery and MRA findings in both groups were analysed with Pearson’s chi-squared test of association. Statistical significance was fixed at *p* < 0.05 for all tests performed.

## Results

Forty-one patients met the inclusion criteria (*n* = 21 in group A, *n* = 20 in group B) (Table 1).

**Table 1:** Intraoperative and arthro-MRI findings for each patient

Patient	X-ray-based FAI classification	Treatment		Cam MRI/intraop	Femoral head chondral lesion MRI/intraop	Os acetabuli MRI/intraop	Labral lesion RMN/intraop	Acetabular chondral lesion RMN/intraop
1	Cam	Arthroscopy		y/y	y/y	n/n	n/n	n/n
2	Cam	Arthroscopy		y/y	n/y	n/n	n/y	y/n
3	Cam	Arthroscopy		y/y	n/n	n/n	n/y	y/y
4	Cam	Arthroscopy		y/y	n/n	n/n	y/y	n/n
5	Local pincer	Arthroscopy		n/n	n/y	y/y	n/y	n/n
6	Cam	Arthroscopy		y/y	y/n	n/n	n/n	y/n
7	Mixed	Arthroscopy		y/y	n/n	n/n	y/y	n/n
8	Mixed	Arthroscopy		y/y	n/n	n/n	y/y	y/y
9	Mixed	arthroscopy		y/y	n/y	n/n	y/y	n/y
10	Local pincer	Arthroscopy		n/n	n/n	y/y	y/n	n/n
11	Cam	Arthroscopy		n/n	y/y	n/n	y/y	n/n
12	Cam	Arthroscopy		y/y	y/n	n/n	y/y	n/n
13	Cam	Arthroscopy		y/y	n/y	n/n	y/y	n/n
14	Cam	Arthroscopy		y/y	n/n	n/n	y/y	n/n
15	Mixed	Arthroscopy		y/y	n/y	n/n	y/y	n/n
16	Cam	Arthroscopy		y/y	y/y	n/n	n/n	n/n
17	Cam	Arthroscopy		y/y	n/n	n/n	y/y	n/n
18	Mixed	Arthroscopy		y/y	y/y	n/n	y/y	y/y
19	Cam	Arthroscopy		y/y	y/n	n/n	y/y	n/n
20	Local pincer	Arthroscopy		n/n	n/n	y/y	y/y	n/n
21	Mixed	Dislocation		y/y	n/n	n/n	n/n	y/y
22	Mixed	Dislocation		y/y	n/n	n/n	y/y	n/y
23	Mixed	Dislocation		y/y	n/y	y/y	y/y	n/n
24	Mixed	Dislocation		y/y	n/y	y/y	y/y	n/n
25	Pincer	Dislocation		n/n	n/n	n/n	y/y	n/n
26	Pincer	Dislocation		n/n	y/n	n/n	n/n	y/y
27	Mixed	Dislocation		y/y	n/n	n/n	y/y	n/y
28	Mixed	Dislocation		y/y	y/y	n/n	y/y	y/y
29	Mixed	Dislocation		y/y	n/n	n/n	y/y	y/y
30	Mixed	Dislocation		y/y	n/n	y/y	y/y	n/n
31	Mixed	Dislocation		y/y	y/y	n/n	y/y	y/y
32	Mixed	Dislocation		y/y	n/n	n/n	y/y	y/y
33	Mixed	Dislocation		y/y	n/n	n/n	y/y	n/y
34	Mixed	Dislocation		y/y	n/n	n/n	y/y	y/y
35	Mixed	Dislocation		y/y	n/n	n/n	y/y	n/n
36	Pincer	Dislocation		n/n	n/y	n/n	y/y	n/n
37	Mixed	Dislocation		y/y	y/n	n/n	y/y	n/y
38	Mixed	Dislocation		y/y	n/n	n/n	y/y	n/n
39	Mixed	Dislocation		y/y	n/n	y/y	y/y	y/y
40	Mixed	Dislocation		y/y	n/n	n/n	n/n	y/n
41	Mixed	Dislocation		y/y	n/n	y/y	y/y	n/n

Group A included 9 females and 12 males with an average age of 23, while group B included 8 females and 12 males with an average age of 25 years.

The time interval between MRA and surgery was less than six months for all patients (range 2–6 months).

Examining the reports of cam deformities, overall sensitivity, specificity, PPV and NPV were found to be 100 %. For lesions of the femoral head cartilage, the sensitivity was 46 %, specificity was 81 %, PPV 55 % and NPV 73 %. For os acetabuli, the sensitivity, specificity, PPV and NPV were all 100 %. For labral tears, the sensitivity was 91 %, specificity was 86 %, PPV was 97 % and NPV was 67 %. For acetabular cartilage injuries, the sensitivity was 69 %, specificity 88 %, PPV 78 % and NPV was 81 % (Table 2).

**Table 2:** Sensitivity, specificity, PPV and NPV are shown for group A and B lesions

	Dislocation	Arthroscopy	All
Cam deformity			
Sensitivity (%)	100	100	100
Specificity (%)	100	100	100
PPV (%)	100	100	100
NPV (%)	100	100	100
Accuracy (%)	100	100	100
*p* value	<0.001	<0.001	<0.001
Femoral head cartilage injury			
Sensitivity (%)	50	44	46
Specificity (%)	82	73	81
PPV (%)	40	57	55
NPV (%)	88	62	73
Accuracy (%)	76	60	68
*p* value	0.172	0.432	0.095
Os acetabuli			
Sensitivity (%)	100	100	100
Specificity (%)	100	100	100
PPV (%)	100	100	100
NPV (%)	100	100	100
Accuracy (%)	100	100	100
*p* value	<0.001	<0.001	<0.001
Labral tear			
Sensitivity (%)	100	81	91
Specificity (%)	100	75	86
PPV (%)	100	93	97
NPV (%)	100	50	67
Accuracy (%)	100	80	93
*p* value	<0.001	0.028	<0.001
Acetabular cartilage injury			
Sensitivity (%)	67	75	69
Specificity (%)	89	88	88
PPV (%)	89	60	78
NPV (%)	67	93	81
Accuracy (%)	76	85	80
*p* value	0.034	0.068	0.04

## Discussion

MRA is a recognized imaging modality for the detection of lesions of the acetabular labrum. In a recent meta-analysis [10] of 16 studies assessing the sensitivity and specificity of MRA, MRA demonstrated a sensitivity of 87 % and specificity of 64 %. In our study, we achieved similar results, with an overall sensitivity of 91 % and a specificity of 86 %. The diagnosis of cartilage delamination in the hip using MR arthrography has proven to be a challenge. The cartilage surfaces of the hip joint are often poorly delineated without arthrography, even when joint effusion is present.

Because of limited joint distensibility, cartilage delamination in the hip is difficult to detect, even with MRA. Mintz et al. [11] reported that non-enhanced MRI showed high accuracy in the evaluation of hip cartilage injuries (with 86–92 % agreement with arthroscopy in terms of the grade of cartilage injury). However, their study demonstrated no cases of cartilage delamination on either MRI or arthroscopy, a radically different finding from ours, Anderson’s [7] (in which the rate was 44 %), and Beck’s study [12] (in which the rate was 38.5 %). Either these were different patient populations or Mintz et al. [11] simply failed to detect cartilage delaminations. Zlatkin et al. [13] detected 9 of 11 lesions (82 %) on MRI as well as indirect MRA, and stated that “conventional MRI evaluation was qualitatively equal to or better than indirect MRA in this evaluation”. Furthermore, Pfirrmann et al. [3] evaluated 44 patients with a cam FAI and observed a sensitivity, specificity, and accuracy of 22–30, 95, and 57–61 %, respectively. Our study confirms poor sensitivity and specificity for cartilage lesions of the femoral head and the acetabulum, suggesting that, at present, MRA possesses only moderate accuracy for detecting cartilage lesions. In particular, partial thickness cartilage lesions, fissures, and nondisplaced flaps can be difficult to identify.

As the presence of cartilage delamination in the hip is correlated with a poorer surgical outcome, we are of the opinion that improvements in radiographic techniques are required so that more precise information can be provided to the surgeon prior to surgery. We feel that researchers should focus on improving MR procedures, such as the dGEMRIC technique (indirect assessment of articular cartilage glycosaminoglycan content by intravenous administration of gadolinium) [14], in order to give reliable preoperative information to the surgeon.

Our study has some limitations. We did not include a control group of healthy subjects, and the sample size was small, which does not permit statistical analysis. Furthermore, since this was a retrospective study, many patients with asymptomatic femoroacetabular impingement were likely not included in the data analysis. This is unavoidable, however: since the patients were selected on the basis of the presence of clinical signs of impingement, our study is likely to have included only patients with established disease. Although the alpha angle was used to quantify the abnormal head–neck junction, other methods of assessing the head–neck junction have been described previously. All seem to be acceptable quantitative methods. In our study, we used alpha-angle measurement because it was the most feasible method with regard to the use of existing tools on the clinical workstation.

MR arthrography provides reliable information about the presence of a cam-type deformity or an os acetabuli. Once again, this study confirms that MRA is an accurate imaging modality for the assessment of labral injury. At present, the role of MR arthrography in evaluating cartilage lesions appears to be limited.
